# Uniportal Video-Assisted Thoracoscopic Anatomic Lung Resection after Neoadjuvant Chemotherapy for Lung Cancer: A Case-Matched Analysis †

**DOI:** 10.3390/cancers16152642

**Published:** 2024-07-25

**Authors:** Marco Andolfi, Elisa Meacci, Michele Salati, Francesco Xiumè, Alberto Roncon, Gian Marco Guiducci, Michela Tiberi, Anna Chiara Nanto, Dania Nachira, Adriana Nocera, Giuseppe Calabrese, Maria Teresa Congedo, Riccardo Inchingolo, Stefano Margaritora, Majed Refai

**Affiliations:** 1 Department of Thoracic Surgery, AOU delle Marche, 60126 Ancona, Italy; michele.salati@ospedaliriuniti.marche.it (M.S.); francesco.xiume@ospedaliriuniti.marche.it (F.X.); alberto.roncon@ospedaliriuniti.marche.it (A.R.); gianmarco.guiducci@ospedaliriuniti.marche.it (G.M.G.); michela.tiberi@ospedaliriuniti.marche.it (M.T.); annachiara.nanto@ospedaliriuniti.marche.it (A.C.N.); majed.refai@ospedaliriuniti.marche.it (M.R.); 2 Department of General Thoracic Surgery, Fondazione Policlinico Universitario ‘A. Gemelli’ IRCCS, Università Cattolica del Sacro Cuore, 00168 Roma, Italy; elisa.meacci@unicatt.it (E.M.); dania.nachira@unicatt.it (D.N.); adriana.nocera@unicatt.it (A.N.); giuseppe93calabrese@virgilio.it (G.C.); mariateresa.congedo@policlinicogemelli.it (M.T.C.); stefano.margaritora@unicatt.it (S.M.); 3 UOC Pneumologia, Dipartimento Neuroscienze, Organi di Senso e Torace, Fondazione Policlinico Universitario ‘A. Gemelli’ IRCCS, 00168 Rome, Italy; riccardo.inchingolo@policlinicogemelli.it

**Keywords:** uniportal video-assisted thoracoscopy surgery (VATS), neoadjuvant chemotherapy, minimally invasive surgery, VATS lobectomy, anatomic lung resection

## Abstract

**Simple Summary:**

In cases of advanced lung cancer after neoadjuvant chemotherapy (nCT), the role of uniportal video-assisted thoracoscopic surgery (U-VATS) is still questionable, with concerns about safety, technical feasibility, and oncological completeness. The aim of this retrospective study was to assess the impact of nCT on patients who had undergone U-VATS anatomic lung resections for lung cancer. We compared the short-term outcomes of 60 patients with case-matched counterparts (treated by surgery alone) selected by propensity score analysis, finding that U-VATS after nCT is a feasible approach with a similar rate of cardiopulmonary complications, length of stay, and readmission when compared with the control group. However, it is still a challenging surgery due to the great technical complexity, which is responsible for the higher incidence of conversion.

**Abstract:**

Background: The advantages of video-assisted thoracic surgery (VATS) are well-recognized in several studies. However, in the cases of advanced lung cancer after neoadjuvant chemotherapy (nCT), the role of VATS is still questionable, with concerns about safety, technical feasibility, and oncological completeness. The aim of this study was to assess the impact of nCT on patients who had undergone uniportal VATS (U-VATS) anatomic lung resections for lung cancer, by comparing the short-term outcomes of patients after nCT with case-matched counterparts (treated by surgery alone). Methods: We performed a retrospective, comparative study enrolling 927 patients (nCT: 60; non-nCT:867) who underwent U-VATS anatomic lung resections from 2014 to 2020 in two centers. Data were collected in a shared database with standardized variables’ definition. Propensity score matching using 15 baseline preoperative patients’ characteristics was performed in order to minimize selection-confounding factors between the two groups, which then were directly compared in terms of perioperative outcomes. Results: After propensity score matching, two groups of 60 patients had been defined. The nCT-group had a higher conversion rate compared to the control group (13.3% vs. 0%, *p* = 0.003) without an increase in operation time or cardiopulmonary complications. In addition, no differences between the two groups were recorded in terms of prolonged air leaks, length of stay, and readmission. Conclusions: U-VATS after nCT is a feasible approach, showing a similar rate of cardiopulmonary complications and length of stay when compared with the control group. However, it remains a challenging surgery due to its great technical complexity as well as the clinical status of the patients.

## 1. Introduction

The primary goals of the surgical management of locally advanced non-small-cell lung cancer (NSCLC) are patient safety and oncological efficacy. The latter can also be achieved thanks to the induction chemo- and radiotherapy, which, either alone or in combination, have been proposed to downstage tumors and improve the completeness of resection and survival [1,2].

To date, amid the improvement of surgical techniques and technological progress in the thoracoscopic field, several studies have recognized the advantages of video-assisted thoracoscopic surgery (VATS) when compared with the open approach for the treatment of NSCLC [3,4,5,6,7,8,9,10,11,12,13,14]. However, in the case of advanced lung cancer after neoadjuvant chemotherapy (nCT), the role of minimally invasive surgery (MIS) still remains questionable, with concerns about safety, technical feasibility, and oncological completeness [15].

Indeed, tumor extension and lymph nodal involvement may critically impact the surgical procedure. Furthermore, as previously reported by other authors [16,17], the toxicity of platinum-based chemotherapy may play a role affecting in the postoperative course, due to subclinical parenchymal and systemic damage, responsible for the fibrosing effect and capillary leak syndrome. All these aspects, together with the vascular fragility and pleural adhesions, make the surgery and the perioperative management a challenge even for an experienced surgeon.

Additionally, only very few retrospective comparative studies, all based on small sample sizes, have focused their attention on the feasibility of the uniportal VATS (U-VATS) approach after nCT [18,19,20], and among these, only one report was conducted on patients who had undergone the same MIS [21].

The aim of this study was to assess the impact of nCT on patients who had undergone U-VATS anatomic lung resections for lung cancer, by comparing the short-term outcomes of those patients with case-matched counterparts (treated by surgery alone) selected by propensity score analysis. Specifically, the conversion rate and cardiopulmonary complications represented the primary outcomes for our comparison between the two groups. The length of stay and readmission after discharge were considered secondary outcomes.

## 2. Materials and Methods

### 2.1. Study Design and Patient Information

This was a retrospective, comparative study enrolling patients who had undergone U-VATS anatomic lung resections in two different Italian thoracic surgical centers that share the same surgical approach, VATS experience, and skills. Surgical procedures were performed by experienced and junior surgeons as well as by residents, according to the complexity of the case. Data were collected in a dedicated dataset including 60 shared variables.

During the study period (2014–2020), we enrolled 927 patients scheduled to undergo elective U-VATS anatomic lung resection at the Departments of AOU delle Marche in Ancona and the Policlinico Universitario ‘A. Gemelli’ in Rome.

Anatomic lung resections included segmentectomy, lobectomy, and bilobectomy for lung cancer. All enrolled patients were informed by medical staff and signed informed written consent for us to use their anonymous data for scientific purposes.

The study population was divided into two groups: the “nCT” group and the “non-nCT” group. Indications for nCT were based on international guidelines and multidisciplinary discussion in accordance with the stages of disease and the patients’ comorbidities. All patients were operated on by the staff surgeons using the same standardized U-VATS approach, as reported in the literature [22]. The definition of each variable was compliant with those reported by the STS and ESTS societies [23] or defined by consensus and shared among the two centers.

The postoperative patient management was generally aligned between the two centers and was based on standardized fast-tracking protocols, as previously described [24,25].

To minimize selection-confounding factors between the two groups (nCT and non-nCT) in relation to the outcomes, we performed propensity score matching. After matching, the outcomes of the nCT-group patients (intervention cohort) were directly compared to those of their counterparts (control cohort).

Patients were considered eligible for inclusion if they were ≥18 years of age, affected by lung cancer treated with nCT consisting of 3 cycles of platinum-based treatment (Table 1), and scheduled for elective U-VATS anatomic lung resection.

Patients were excluded from the analysis for any of the following reasons: undergoing pneumonectomy, benign/non-neoplastic/metastatic disease, operated through the open approach, lung cancer treated with neoadjuvant radiotherapy, targeted therapy, and/or neoadjuvant immunotherapy, considering that the latter treatments could influence the surgical outcomes due to worse results reported in terms of tissue quality, adhesions, inflammation, or fibrosis compared to the standard platinum-based treatment [21].

This study involving human participants was reported to the local ethical committee for its examination (n. 2022-11) even though, due to the retrospective nature of the work, formal approval was not required.

### 2.2. Outcomes

In order to evaluate the impact of nCT on U-VATS patients, we selected and measured the following parameters: length of surgical procedure, conversion rate, postoperative cardiopulmonary complication rate, prolonged air leak (PAL, if >5 days), length of stay, permanence of chest tube, and in-hospital readmission rate. We considered the following cardiopulmonary complications with standardized definitions as reported in the literature [23]: pneumonia, atelectasis requiring bronchoscopy, respiratory failure, reintubation, acute respiratory distress syndrome, pulmonary embolism, pulmonary edema, acute myocardial ischemia, arrhythmia, acute cardiac failure, stroke or transient ischemic attack, and acute kidney disease. We evaluated the complications that arose within the first 30 days after the operation and/or during the entire hospital stay. Readmission was defined as any new patient hospital admission within 30 days from discharge. Conversion to open was defined as operations that began with uniportal VATS dissection of hilar structures and finished as rib-spreading lateral muscle-sparing thoracotomy.

### 2.3. Statistical Analysis

Data for the present analysis were retrieved from the two databases respectively maintained by each institution participating in this study. Data were merged using a matching grid, assuring the correspondence of the variables selected for this study.

Before analysis, data underwent data quality assessment and data cleaning procedures. Variables with a completeness rate <90% and patients with missing data for one of the selected outcomes were excluded from the analysis. Specific imputation techniques were used to clean variables with incompleteness up to 10%. The 15 variables included in the present analysis and their completeness are reported in Table 2.

In this study, a wide number of baseline characteristics with a potential impact on postoperative outcomes were used to match the patients, to increase the reliability of the comparison between the two groups (Table 2).

Because of the necessary imbalance, clinical staging, reported in Table 3, was not used in the construction of the propensity model.

Numeric variables were assessed for normality by the Shapiro–Wilk test. The unpaired Student *t* test was used to test numeric variables with a normal distribution, and the Mann–Whitney test was used for those without a normal distribution. The chi-square test was used to test categorical variables.

Propensity score matching was used to reduce the effects of potential confounding factors between the nCT and non-nCT groups in relation to the outcomes. According to clinical and scientific knowledge, the following variables were included in the propensity score model, which were potentially linked to exposure to nCT, the outcome, or both: age, sex, American Society of Anesthesiologists (ASA) score, Eastern Cooperative Oncology Group score, chronic obstructive pulmonary disease index, body mass index, forced expiratory volume in 1 s (FEV1), Tiffeneau (TIFF) index, carbon monoxide diffusing capacity (DLCO), coronary artery disease, hypertension (HT), cerebrovascular disease, chronic kidney disease, chronic liver disease, and type of resection (lobectomy, bilobectomy, segmentectomy). After the propensity score creation, patients were matched based on the propensity score in a 1:1 ratio. As recommended [26], calipers of a width of 0.2 of the standard deviation of the logit of the propensity score were used. The standardized differences mean (SDM) was estimated after matching, to evaluate the balance of covariates, with absolute values greater than 0.1 indicative of imbalance between groups. A *p*-value less than 0.05 was considered statistically significant. Statistical analysis was performed on the statistical software Stata 14.0 (StataCorp, LP, College Station, TX, USA).

## 3. Results

All 927 enrolled patients (mean age 68.6, 43% males) were treated with the U-VATS approach. Before matching, the two groups of patients (nCT vs. non-nCT), consisting of 60 and 867 patients, respectively, showed several statistically significant differences in terms of preoperative patients’ characteristics, involving age (63.7 vs. 68.9, *p* < 0.001), ASA score (2 vs. 1.61, *p* < 0.001), and HT (35% vs. 56.2%, *p* = 0.001) (Table 2). However, after matching, the two groups became homogeneous (Table 2) and were directly compared.

After matching, adenocarcinoma was the most common histology (65%), followed by squamous cell carcinoma (24%) and neuroendocrine carcinoma (6%, see Table 3). Fifty (83%) treated patients and fifty-three (88%) control group patients were in the early stage (0-II stage, according to AJCC, VIII edition) at the final pathological finding.

From the comparison, we found that the nCT-group had a higher conversion rate compared to the control group (13.3% vs. 0%, *p* = 0.003) without an increase in the operation time (200.4 min vs. 206 min, *p* = 0.66) or the cardiopulmonary complication rate (13.3% vs. 13.3%, *p* = 1, see Table 4).

The causes of conversion to open were bleeding (three patients), metastatic lymph nodes (three patients), and severe adhesions (two patients). All patients who required conversion to open underwent a lobectomy (four left lower lobectomy, two right lower lobectomy, two left upper lobectomy). Furthermore, no statistically significant differences were recorded in terms of PAL (13.3% vs. 6.7%, *p* = 0.22), chest tube days (4.6 vs. 3.6, *p* = 0.2), length of stay (5.1 vs. 4.8 days, *p* = 0.69), and readmission rate (5% vs. 10%, *p* = 0.29) (Table 4). Hydropneumothorax was the most common cause of readmission (5/9 patients), followed by wound infection and pneumonia.

Finally, no differences were recorded in the number of lymph node stations (3.6 in the nCT group vs. 3.9 in the non-nCT group, *p* = 0.23) or the lymph nodes retrieved during lymphadenectomy (12.5 ± 7.6 in the nCT group vs. 10.6 ± 2.8 in the non-nCT group, *p* = 0.06, see Table 4).

All matched patients received a complete R0 resection. Only one nCT patient needed reoperation for hemothorax.

## 4. Discussion

In this study, nCT did not negatively impact the postoperative course of patients who underwent U-VATS resection, guaranteeing the same cardiopulmonary complication rate (13.3% vs. 13.3%, *p* = 1) for both groups and a similar length of stay (5.1 vs. 4.8 days, *p* = 0.69) and readmission rate (5% vs. 10%, *p* = 0.29). However, it played a significant role in the intraoperative phase, when it was responsible for a higher incidence of conversion to open (13.3% vs. 0%, *p* = 0.003).

Limitations of this study include (1) the retrospective nature; (2) the limited number of patients, with a consequent potential weakness in terms of the results’ generalizability (therefore, to reduce the effects of potential confounding factors between the n-CT and non-nCT groups in relation to the outcomes, propensity score matching was performed); (3) potential minor discrepancies in the postoperative management between the two groups (these were inevitable due to the nature of this study, even if the U-VATS approach was the same as that reported in the literature [22] and the perioperative patient management was aligned between the two centers, based on standardized fast-tracking protocols defined by consensus, and shared among the two centers); (4) being an observational study performed with patients referred from various local regional hospitals, which meant it was not possible to retrieve detailed information about pre-treatment clinical stages, chemotherapy protocols, patients’ compliance, or complications related to the treatment; and (5) the lack of a comparison group of open surgery or other VATS approaches.

To date, only one retrospective comparative study has been conducted on patients who underwent the same MIS [21], reporting the short-term results of U-VATS performed for patients with NSCLC after nCT. They concluded that U-VATS after nCT was a feasible and relatively safe approach, showing significant differences only in terms of parenchymal fistula (5.1% vs. 22.2%, *p* = 0.013) and reoperation rates (3.7% vs. 22.2%, *p* = 0.007), which were higher in the 18 patients who received nCT.

Considering the previous preliminary results, and with the intention of testing their generalizability, we performed the present study, aimed at verifying the early postoperative outcomes in patients undergoing U-VATS anatomic lung resection after nCT in a larger cohort and using a propensity score matching comparison versus their non-chemo-treated counterparts.

Our results confirmed that U-VATS was a feasible approach even for the patients who were treated with nCT. The higher rate of conversion to open in the nCT-group, even though it did not differ from those already published for other minimally invasive techniques [11,21], was an index of the greater technical complexity related to the clinical consequences of the treatment received rather than an index of the local extension of the disease. Indeed, in our series, considering that 50/60 patients after treatment (83%) were in the early stage (0-II stage), and among these, 7 patients were ypT0N0, we can state that technical difficulties were mostly related to tissue quality, adhesions, inflammation, and fibrosis rather than to the local extension of the disease.

Nevertheless, the different conversion rates did not impact the operation times, which overlapped between the two groups. We should consider that the surgical procedures were performed by experienced and junior surgeons as well as by residents, and this could play a role when considering the optimization of the operation time. However, we did not consider it as a bias of this study because there was a similar distribution of experienced and junior surgeons in the two groups (junior surgeons in nCT-group 14.3% vs. 15% in control group, *p* = 0.92).

Additionally, unlike in other studies where the parenchymal fistula was the most common minor complication after nCT both in VATS and in the open approach [21,27], here, PAL was similar in the two groups (13.3% vs. 6.7%, *p* = 0.22), despite the tissue adhesions and the fibrosing effect on the pulmonary parenchyma, caused by the platinum-based nCT. Indeed, nCT can provoke diffuse interstitial alteration, a combination of subacute and chronic lung involvement with remodeling of the alveolar septa, and an increase in the content of proteins of the extracellular matrix responsible for the development of fibrotic involvement [7,17,28].

In this study, the length of stay, the chest tube duration, and the average number of lymph nodes removed were comparable and in line with those reported in the literature [7,28,29].

Although the advantages of MIS over the open approach for the treatment of early-stage NSCLC have been well recognized by several studies, the role of MIS after nCT still remains debatable [15]. In fact, due to the lack of prospective studies, VATS could offer an alternative after nCT, but only for selected patients with small, peripheral, weakly positive N2, and non-squamous NSCLC. However, there is still limited high-level evidence to support the notion that it should be the standard treatment for this kind of patient [15].

On the other hand, amid the development and diffusion of minimally invasive techniques, the safety and feasibility of VATS following nCT have been increasingly reported, providing better results in terms of surgical outcomes when compared with the open approach without worsening the long-term oncological results [3,4,5,6,7,8,9,10,11,12,13,14,18,19,20,21].

Anyway, according to the literature, patients undergoing nCT experienced more postoperative complications than others who underwent surgery alone. In particular, the incidences of cardiovascular and respiratory diseases (38.8% vs. 30.9% and 44.4% vs. 30.1%, respectively) were higher in treated patients [30].

## 5. Conclusions

In conclusion, to the best of our knowledge, this is the largest comparative study describing the short-term results of U-VATS anatomical lung resection performed for lung cancer with or without nCT, conducted on patients who had undergone the same MIS.

Taking into account the higher rate of conversion to open, we surmise that U-VATS after nCT is a challenging surgery due to its greater technical complexity related to the treatment. However, it represents a feasible approach, showing the same cardiopulmonary complication rate and a similar length of stay and readmission rate for both groups.

Understandably, extensive experience, a solid foundation in VATS, and rational standardization of surgical procedures are crucial aspects in guaranteeing the successful completion of U-VATS.

Finally, due to the limitations previously described, the lack of prospective evidence, and the heterogeneity of patients reported in the literature, including few resections and different chemotherapy protocols, further large-scale studies are needed in the future to validate these preliminary results.

## Figures and Tables

**Table 1:** Different neoadjuvant platinum-based protocols adopted.

nCT	Patients (*n*)
Cisplatin + Pemetrexed	10
Carboplatin + Pemetrexed	5
Carboplatin + Paclitaxel	12
Cisplatin + Gemcitabine	33

**Table 2:** Patients’ baseline characteristics before and after matching.

Variable	Before Matching				After Matching			
	nCT (60)	Non-nCT (867)	*p*-Value	SDM	nCT (60)	Non-nCT (60)	*p*-Value	SDM
Age, y, mean (SD)	63.7 (9.3)	68.9 (10.2)	<0.001	−0.53	63.7 (9.3)	65.4 (12.0)	0.4	0.10
Sex, Male, *n* (%)	29 (48.3%)	366 (42.2%)	0.35	−0.12	29 (48.3%)	29 (48.3%)	1	<0.01
ASA, mean (SD)	2 (0.71)	1.6 (0.61)	<0.001	0.60	2 (0.71)	2.02 (0.72)	0.89	0.03
ECOG, mean (SD)	1.5 (0.57)	1.42 (0.61)	0.32	0.14	1.5 (0.57)	1.43 (0.72)	0.57	0.10
COPD, mean (SD)	1.63 (0.82)	1.51 (0.78)	0.23	0.15	1.63 (0.82)	1.48 (0.77)	0.3	0.10
BMI, mean (SD)	25.8 (4.9)	26.4 (4.7)	0.34	−0.12	25.8 (4.9)	26.2 (4.5)	0.6	0.08
FEV1, mean (SD)	91.1 (21.9)	90.5 (20.1)	0.82	0.03	91.1 (21.9)	93.8 (20.8)	0.5	0.10
TIFF, mean (SD)	0.73 (0.11)	0.72 (0.12)	0.98	0.09	0.73 (0.11)	0.74 (0.13)	0.04	0.08
DLCO, mean (SD)	70,9 (22.4)	74,1 (17.7)	0.17	−0.16	70.9 (22.4)	72.5 (18.1)	0.67	0.08
CAD, yes, *n* (%)	4 (6.47%)	83 (9.57%)	0.45	−0.11	4 (6.67%)	8 (13.33%)	0.22	0.10
HT, yes, *n* (%)	21 (35%)	487 (56.2%)	0.001	−0.43	21 (35%)	18 (30%)	0.56	0.10
CVD, yes, *n* (%)	3 (5%)	74 (8.54%)	0.34	−0.12	3 (5%)	3 (5%)	1	<0.01
CKD, yes, *n* (%)	4 (6.67%)	113 (13%)	0.15	−0.20	4 (6.67%)	4 (6.67%)	1	<0.01
CLD, yes, *n* (%)	6 (10%)	85 (9.80%)	0.96	0.01	6 (10%)	3 (5%)	0.29	0.10
Type of Resection, *n* (%) Lobectomy Bilobectomy Segmentectomy	54 (90%) 1 (1.7%) 5 (8.3%)	708 (81.6%) 12 (1.4%) 147 (17%)	0.22	0.23	54 (90%) 1 (1.7%) 5 (8.3%)	48 (80%) 0 (0%) 12 (20%)	0.12	0.10

nCT: neoadjuvant chemotherapy, SD: standard deviation, ASA: American Society of Anesthesiologists, ECOG: Eastern Cooperative Oncology Group, COPD: chronic obstructive pulmonary disease, BMI: body mass index, FEV1: forced expiratory volume in 1 s, TIFF: Tiffeneau index, DLCO: carbon monoxide diffusing capacity, CAD: coronary artery disease, HT: hypertension, CVD: cerebrovascular disease, CKD: chronic kidney disease, CLD: chronic liver disease.

**Table 3:** Clinical and pathological characteristics of nCT-treated patients and matched controls.

Variable	After Matching	
	nCT (60)	Non-nCT (60)
Histology		
Adenocarcinoma, *n* (%)	37 (61.6)	41 (68.3)
Squamous cell carcinoma, *n* (%)	20 (33.3)	9 (15)
Neuroendocrine carcinoma, *n* (%)	-	7 (11.6)
Other, *n* (%)	3 (5)	3 (5)
cTNM		
T1N0M0	-	40
T1N0M1	3	-
T1N1M0	1	-
T1N2M0	5	1
T1N3M0	2	-
T2N0M0	-	11
T2N0M1	1	-
T2N1M0	4	2
T2N2M0	12	3
T3N0M0	1	1
T3N1M0	3	-
T3N2M0	15	-
T4N0M0	-	2
T4N0M1	5	-
T4N1M0	2	-
T4N2M0	6	-
(y)pTNM		
T0N0M0	7	-
T1N0M0	17	32
T1N1M0	2	-
T1N2M0	5	-
T2N0M0	15	16
T2N1M0	5	1
T2N2M0	1	5
T3N0M0	4	4
T3N1M0	1	-
T3N2M0	1	-
T4N0M0	2	1
T4N1M0	-	1
Clinical stage		
I	-	50
IIA	-	1
IIB	6	3
IIIA	30	6
IIIB	15	-
IV	9	-
Pathological stage		
0	7	-
I	25	47
IIA	7	1
IIB	11	5
IIIA	9	7
IIIB	1	-

**Table 4:** Intervention effect outcomes for nCT-treated patients and matched controls.

Variable	After Matching		
	nCT (60)	Non-nCT (60)	*p*-Value
Operation time, min, mean (SD)	200.4 (78.9)	206.0 (63.7)	0.66
Conversion to open, *n* (%)	8 (13.3%)	0 (0%)	0.003
Total lymph nodes retrieved, *n* (SD)	12.5 (7.6)	10.6 (2.8)	0.06
Total lymph node stations, *n*	3.6	3.9	0.23
Cardiopulmonary complications, *n* (%)	8 (13.3%)	8 (13.3%)	1
Prolonged air leak, *n* (%)	8 (13.3%)	4 (6.7%)	0.22
Chest tube days, d, mean (SD)	4.6 (4.1)	3.6 (4.2)	0.20
Length of stay, d, mean (SD)	5.1 (3.3)	4.8 (3.8)	0.69
Readmission rate, *n* (%) Hydropneumothorax, *n* Wound infection, *n* Pneumonia, *n*	3 (5%) 2 1 -	6 (10%) 3 1 2	0.29

nCT: neoadjuvant chemotherapy, SD: standard deviation.

## Data Availability

The data underlying this article will be shared on reasonable request to the corresponding author.
